# Impact of meteorological parameters and air pollutants on airborne concentration of *Betula* pollen and Bet v 1 allergen

**DOI:** 10.1007/s11356-023-29061-z

**Published:** 2023-08-07

**Authors:** Jana Ščevková, Jozef Dušička, Eva Zahradníková, Regina Sepšiová, Jozef Kováč, Zuzana Vašková

**Affiliations:** 1 Department of Botany, Faculty of Natural Sciences, Comenius University, Révová 39, 811 02 Bratislava, Slovakia; 2 Department of Genetics, Faculty of Natural Sciences, Comenius University, Ilkovičova 6, 842 15 Bratislava, Slovakia; 3 Department of Applied Mathematics and Statistics, Faculty of Mathematics, Physics and Informatics, Comenius University, Mlynská Dolina, 842 48 Bratislava, Slovakia

**Keywords:** Bet v 1 allergen, Birch pollen, Allergenicity of pollen, Air pollutants, ELISA assay, Cyclone sampler

## Abstract

The intensity of birch pollen season is expressed by seasonal pollen integral (SPIn, the sum of the mean daily pollen concentration during the birch pollination period) and the amount of Bet v 1 allergen released per birch pollen grain expressed by pollen allergen potency (PAP). *Betula* pollen and Bet v 1 allergen were simultaneously measured in the air of Bratislava from 2019 to 2022 by using two Burkard traps (Hirst-type and cyclone) in order to evaluate the causes of the seasonal variation in the SPIn and PAP levels. The highest SPIn (19,975 pollen/m^3^) was observed in 2022 and the lowest one (1484 pollen/m^3^) in 2021. The average daily PAP level (4.0 pg Bet v 1/pollen) was highest in 2019 and lowest (2.5 pg Bet v 1/pollen) in 2020. We found that seasonal variation in SPIn was associated mainly with the changes in environmental conditions during the pre-season period, whereas the year-to-year variation in PAP levels was attributed to environmental conditions during both pre- and in-season periods. Our results indicate that rainy weather in June 2020 and cold overcast weather in January‒February 2021 resulted in low SPIn in 2021. On the other hand, dry weather in June 2021 and warm weather in January‒February 2022 resulted in high SPIn in 2022. The low average daily PAP level in 2020 was associated with (1) low levels of gaseous air pollutants in March, when the ripening of pollen takes place; (2) an earlier start of the birch main pollen season (MPS); and (3) dry weather during the MPS. On the other hand, high PAP level in 2019 was associated with higher levels of air pollutants in March and during the MPS.

**Supplementary Information:**

The online version contains supplementary material available at 10.1007/s11356-023-29061-z.

## Introduction

Birch pollen grains elicit allergic inflammation reactions in more than 100 million people worldwide (Lavaud et al. 2013). In Slovakia, two main Betula species (*B.pendula* and *B. pubescens*) occur naturally. Besides natural habitats, birch, especially *B. Pendula*, is frequently planted as an ornamental tree. In central Europe, *B. pendula* is among the main arboreal plant species responsible for pollinosis in spring (Biedermann et al. 2019). Birch trees are anemophilous and produce a great number of pollen grains (1.7 million pollen grains per catkin) (Ranpal et al. 2022) with appropriate aerodynamic properties (Skjøth et al. 2007). It is, therefore, one of the most abundantly present pollen aeroallergen in Bratislava, representing more than 20% of the total captured pollen per annum (Ščevková et al. 2010).

Three important allergens, Bet v 1, 2, and 4, are present in *Betula* pollen (Ciprandi et al. 2016). Bet v 1, a protein of 17 kDa molecular weight, belonging to the PR-10 subfamily, is a major allergen of *B*. *pendula* (syn. *B*. *verrucosa*) pollen. Up to 95% of patients sensitized to birch have been reported to respond to Bet v 1 (Kleine-Tebbe et al. 2017), with allergic rhinoconjunctivitis and asthma as the typical symptoms. Airborne intact birch pollen grains are considered primary allergen-bearing particles of Bet v 1 protein, which is located predominantly in the starch granules and partly in the pollen cover layers (exine and intine) (El-Ghazaly et al. 1996), whereas sub-pollen particles (SPPs), formed by fragmentation of pollen grains, are considered secondary allergen carriers. Intact birch pollen grains with a diameter of up to 25 µm are too huge to penetrate deep inside the lungs and therefore are retained in the upper airways, while SPPs of 2.5 µm or less can distribute allergens into the lower airways and even overcome the lung barrier and penetrate the bloodstream (Buters et al. 2010).

Except for the size of the allergen-bearing particles, the seriousness of allergic manifestations depends on the intensity and duration of aeroallergen exposure. The birch pollen season is short and intense. On average, it lasts 24 days in Bratislava and the mean daily pollen concentration and SPIn reach a value of 313 and 7170 pollen/m^3^, respectively (2002–2022, pollen data of the Comenius University in Bratislava). During most days of the pollen season, the daily concentration of birch pollen in the air exceeds the threshold of 90 pollen/m^3^, provoking severe allergy symptoms (Rapiejko et al. 2007).

The prevalence of seasonal respiratory pollen-related allergic diseases (pollinosis) is increasing in the last few decades, especially in densely populated urban areas, which are characterized by higher levels of air pollution (World Health Organization 2021). Air pollutants, mainly those related to heavy traffic, alter the allergenicity of pollen grains and the patient’s susceptibility to them, which is manifested by an increase in the number of sufferers and/or exacerbation of pollinosis symptoms in cities (Schiavoni et al. 2017). Gaseous pollutants like ozone, carbon monoxide, nitrogen dioxide, and sulfur dioxide and solid particulates (fractions PM_2.5_ and PM_10_) can modify the morphological structure of the outer envelope of pollen grains (exine) or aggravate their allergenicity by increasing the expression or prompting the secretion of new types of allergenic proteins (Visez et al. 2020; Zhou et al. 2021). The pollutants adhered to pollen can damage its surface and cause an enhanced release of allergens (Behrendt et al. 1997) or form pollen-particle complexes due to the coagulation process (Schiavoni et al. 2017). Simultaneously, increased expression of allergenic proteins in plants is a part of their defense mechanism against abiotic stress with pollutants being one of the major stressors (Midoro-Horiuti et al. 2001).

To suppress the COVID-19 pandemic, which hit Slovakia at the beginning of 2020, the Slovak government has implemented various measures, such as suspending industrial production and traffic restriction to limit the transmission of the SARS-CoV-2 virus. The stringent lockdown measures from March 16, 2020 to June 14, 2020 resulted in a significant improvement of air quality in Bratislava in spring 2020 (Ščevková et al. 2021), just as in many other cities worldwide (e.g., Venter et al. 2020; Li et al. 2022). However, several researchers (Hasnain et al. 2021; Gopikrishnan et al. 2022) noted that due to the ease of anti-pandemic restrictions since the second half of 2020, the air quality gradually deteriorated and the levels of major pollutants increased in the air of different parts of the world. From the second half of 2021 onwards, energy prices have been rising sharply in Europe and globally as a consequence of the global pandemy. Moreover, since February 24, 2022 as a result of Russian aggression against Ukraine, fuel prices have increased even further. In this context, it is essential to note that the higher the price of fuel, the lower the concentration of air pollutants (Barnett and Knibbs 2014; Raeissi et al. 2022).

This study aimed to find out whether and how the weather conditions and air pollutants during the pre-season and in-season pollen period affect the intensity of birch MPS, based on average daily concentrations of birch pollen and Bet v 1 allergen measured in Bratislava over 4 years (2019 − 2022).

## Materials and methods

### Study area

The research study was conducted in Bratislava, situated in the Southwestern part of Slovakia, characterized by a moderate to warm continental climate. The average daily temperature is 10.7 °C and yearly precipitation total 655.7 mm (1983‒2018 average, Meteorological observatory Mlynská dolina in Bratislava). Pollen and allergen sampling was carried out daily from February 1 to May 31 over 4 consecutive years, 2019–2022 (start and end time: 10 a.m.). The samplers were located in Bratislava on the Science Park Comenius University building roof (48.149444° N, 17.073333° E, 18 m above the ground).

Birches have been abundantly planted in Slovak cities as an ornamental tree. Its cultivation is being, however, gradually abandoned, especially in the vicinity of residential buildings and schools, replaced by other, less allergenic trees. In Bratislava, birches are planted mainly along the roadsides, in parks, gardens, and other green spaces. Even though it is a component of almost all woodland communities, it is only sporadically present in forests in the surrounding of Bratislava.

### Aerobiological analysis

To evaluate the airborne birch pollen and Bet v 1 concentrations, the Hirst-type Burkard pollen trap and Burkard cyclone sampler were used, respectively. In the Hirst-type pollen trap, pollen grains are attached to adhesive melinex tape. After exposure, the tape was divided into segments, representing 24 h of samplings, and mounted on microscopic slides. The evaluation process followed the methodology described in detail by Galán et al. (2007).

To evaluate the microscopic slides in order to determine the average daily pollen concentration in the air (pollen/m^3^), we used the method of twelve vertical transects at × 400.

The samples from the multi-vial cyclone sampler (pollen grains and SPPs separated in a small cyclone and captured into Eppendorf vials, each representing 24 h exposure) were evaluated at the end of the birch pollen season after previous storage at – 20 °C. The extraction procedure of Bet v 1 allergen was implemented according to the methodology described by Plaza et al. (2016).

The concentration of Bet v1 allegren in samples was determined by enzyme linked immunoabsorbent assay (ELISA) using Bet v 1 2.0 EP kit (Indoor Biotechnologies). The procedure was performed according to manufacturer instructions. Measurement of final absorbance values was carried out at 450 nm with the use of HiPo Microplate Photometer MPP-96 (Biosan). Daily average concentrations of Bet v 1 were stated in pg/m^3^.

The birch main pollen season (MPS) was defined following the 90% method proposed by Nilsson and Persson (1981). The dates of accumulating 5% and 95% of the total yearly birch pollen concentration were taken as the start and end of the birch MPS, respectively. The peak date splits the MPS into pre-peak and post-peak periods.

The following characteristics of the MPS were analyzed: start, end, duration, peak pollen value, and date and SPIn. Besides the above mentioned pollen-related characteristics, we also analyzed allergen-related characteristics of the MPS as follows: peak allergen value and date and seasonal allergen integral (SAIn, the sum of the mean daily allergen concentration over the MPS).

Birch PAP values were calculated as the ratio of the daily Bet v 1 allergen and birch pollen concentrations, given in pg Bet v 1/pollen.

### Environmental parameters

The meteorological data were supplied by the Meteorological Observatory Mlynská dolina in Bratislava (48.151111°N, 17.070556°E), close to the aerobiological station. The meteorological variables analyzed in daily resolution were mean air temperature (°C), total sunshine (hours), mean relative humidity (%), and total precipitation (mm).

The air pollution data were supplied by the Slovak Hydrometeorological Institute. The pollutants (in μg/m^3^) analyzed daily were particulates (PM_10_ and PM_2.5_); O_3_, ozone; CO, carbon monoxide; and NO_2_, nitrogen dioxide.

### Data analysis

To measure the strength of association between pollen and allergen concentrations as well as between pollen and PAP levels, nonparametric Spearman correlation test was used.

SPIn of birch pollen depends mostly on pollen production, which is affected by meteorological conditions and air pollutants at different stages of plant development (Piotrowska and Kubik-Komar 2012; Grewling et al. 2012), especially the month of the previous year after the end of the birch pollen season when male catkins containing microspores are formed (June in temperate climate) and the period after dormancy in the current year when the development of pollen grains takes place (January–February). PAP of birch pollen in a particular pollen season depends most on the air pollution at the time of the final maturation of pollen in anthers (approximately 1 week before pollen liberation), as this period is associated with the start of secretion of allergenic molecules (Buters et al. 2010). This week usually occurs during March in the pre-season period. Before this time, no allergens were detected in pollen (Oh 2022). The synthesis of allergens in pollen grains, once they have been released from anthers, is also unlikely since pollen desiccates immediately outside of the anther, leading to a metabolism drop (Pacini and Dolferus 2019) until landing on a receptive stigma. This implies that the content of allergenic molecules in pollen grains outside the anthers does not change significantly (Sharif Shoushtari et al. 2013). To evaluate the effect of selected environmental parameters during the pre-season periods on in-season birch SPIn or PAP levels, we assessed the average values of the 4 studied years (2018–2021 for environmental parameters during the year preceding pollination and 2019–2022 for parameters during the year of pollination, SPIn and PAP). To establish comparability among the average values of individual variables, a standardization process was employed before evaluation. Specifically, for each value within a variable, the 4-year average was subtracted, followed by division by the 4-year standard deviation. This standardized the values, thereby ensuring that the observed averages would reside on a uniform scale.

The discharge of matured pollen grains from microsporangia and its air dispersal are influenced by environmental factors during the MPS. To measure the impact of the meteorological parameters and air pollutants on the pollen, allergen, and PAP levels over the birch MPS, we applied multiple regression analyses. Time lags up to 6 days were considered for environmental variables. For each variable and each model, the lag that was most correlated with the response variable was selected in the model. To account for differences between years, a factor variable “year” (with levels 2019, 2020, 2021, and 2022) was included in the models in addition to the environmental variable. A logarithmic transformation of the pollen and PAP variables was applied to achieve normality of the residuals. To account for the autocorrelation, we applied the ARMA correlation structure to the covariance matrix of the residuals, with each year represented by a separate block (Pinheiro and Bates 2000). To fit regression models that included all the explanatory variables for pollen, allergen, and PAP levels, the gls function from the nlme package was used. To eliminate the variables with a *p*-value less than 0.05 from the model, the backward elimination approach was used.

## Results

Table 1 shows the characteristics of individual birch pollen seasons and Bet v 1 allergen occurrence over the analyzed years. The year 2021 had 14-fold less birch SPIn (1484 vs 19,975 pollen*day/m^3^) and sixfold less Bet v 1 SAIn (3652 vs 20,381 pg/m^3^) than 2022, showing a significant seasonal variability in pollen, and subsequently allergen production.

**Table 1:** Main pollen-season-related characteristics of *Betula* pollen seasons in Bratislava, years 2019 − 2022

Characteristics	2019	2020	2021	2022
*Betula* pollen				
Pollen season start	April 1	March 22	April 1	March 29
Pollen season end	April 25	April 24	May 5	May 1
Season length (days)	25	34	35	34
Seasonal pollen integral (pollen/m^3^)	6478	8402	1484	19,975
Peak value (pollen/m^3^)	945	1035	285	4899
Peak date	April 9	March 29	April 22	April 7
Mean daily value (pollen/m^3^)	259	247	42	588
Bet v 1 allergen				
Seasonal allergen integral (pg/m^3^)	8770.9	12,974.6	3652.2	20,380.7
Peak value (pg/m^3^)	775.6	978.8	364.8	874.7
Peak date	April 8	March 30	April 22	April 7
Mean daily value (pg/m^3^)	350.8	244.8	104.3	599.4
Minimum daily value (pg/m^3^)	42.0	9.7	22.1	40.3
Pollen allergen potency (pg/pollen)*	4.0	2.5	3.8	3.7
Pollen allergen potency (pg/pollen)**	26.5	8.7	13.2	17.1

^*^Mean daily value; **maximum daily value

**Fig. 1:**
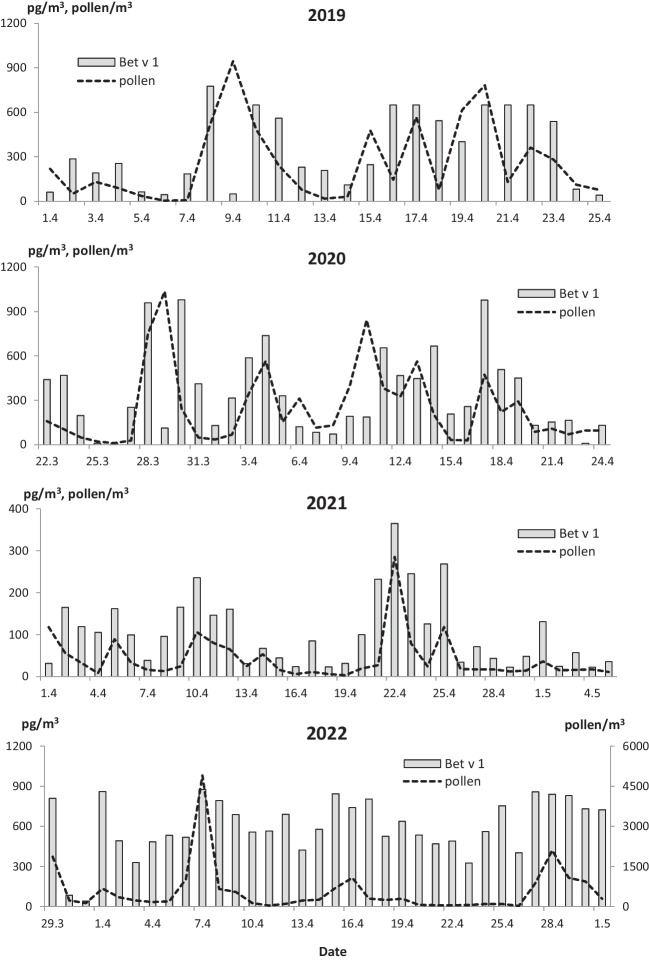
The airborne levels of *Betula* pollen and Bet v 1 allergen in Bratislava over the main polen seasons, years 2019–2022

**Fig. 2:**
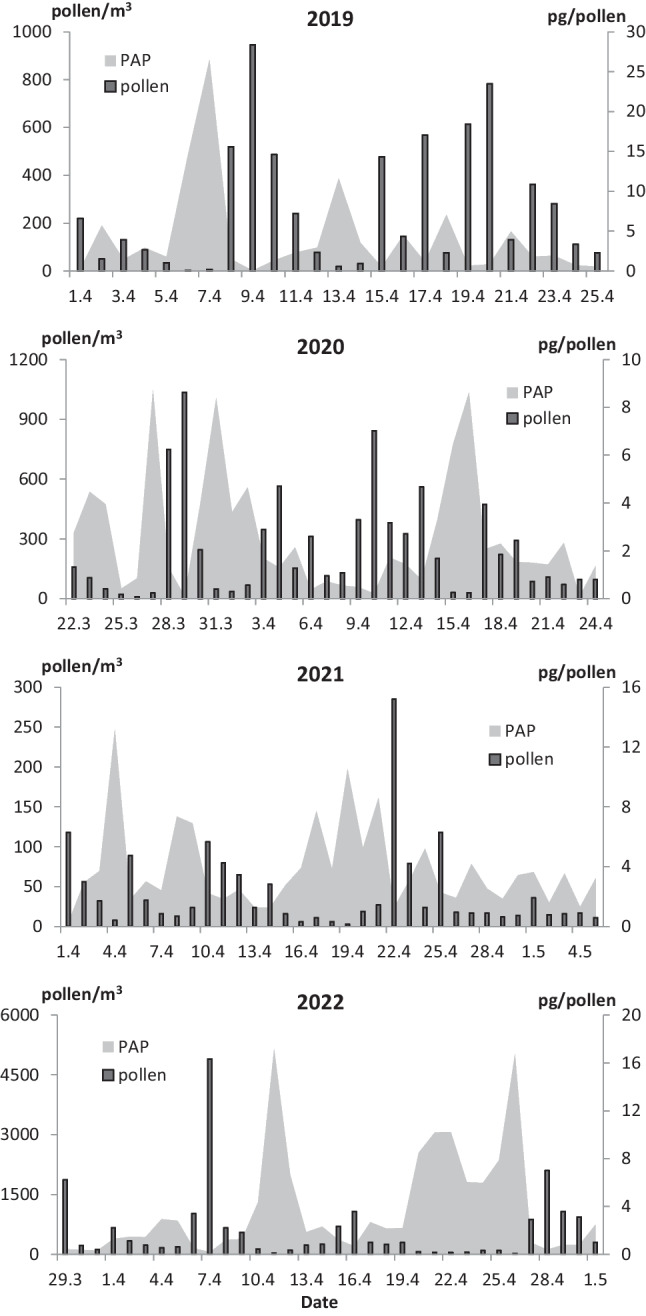
Mean daily birch pollen concentrations and PAP levels over the birch main pollen seasons

Airborne *Betula* pollen and Bet v 1 allergen were present in the study area since the end of March to the beginning of May (Fig. 1). Considering the timing, the earliest start of birch MPS was in March 22, 2020, which is more than 1 week earlier than in the other analyzed years. Each year, pollen and allergen levels reached peak values almost simultaneously; however, our data hint at some seasonal differences. Our research also revealed year-to-year fluctuations in the peak dates of PAP (Fig. 2).

The results of Spearman’s correlation analysis over the 4 analyzed years revealed statistically significant positive relationships between the birch pollen and Bet v 1 levels, especially in the pre-peak. The correlation coefficients for the pre-peak, MPS, and post-peak periods were 0.691 (*p* ˂ 0.001), 0.456 (*p* ˂ 0.01), and 0.354 (*p* ˂ 0.05), respectively. On the other hand, a significant negative association between pollen and PAP levels (*r* =  − 0.653, *p* ˂ 0.001), manifested by rising PAP levels during days with low pollen concentrations and vice versa, was observed (Fig. 2).

**Fig. 3:**
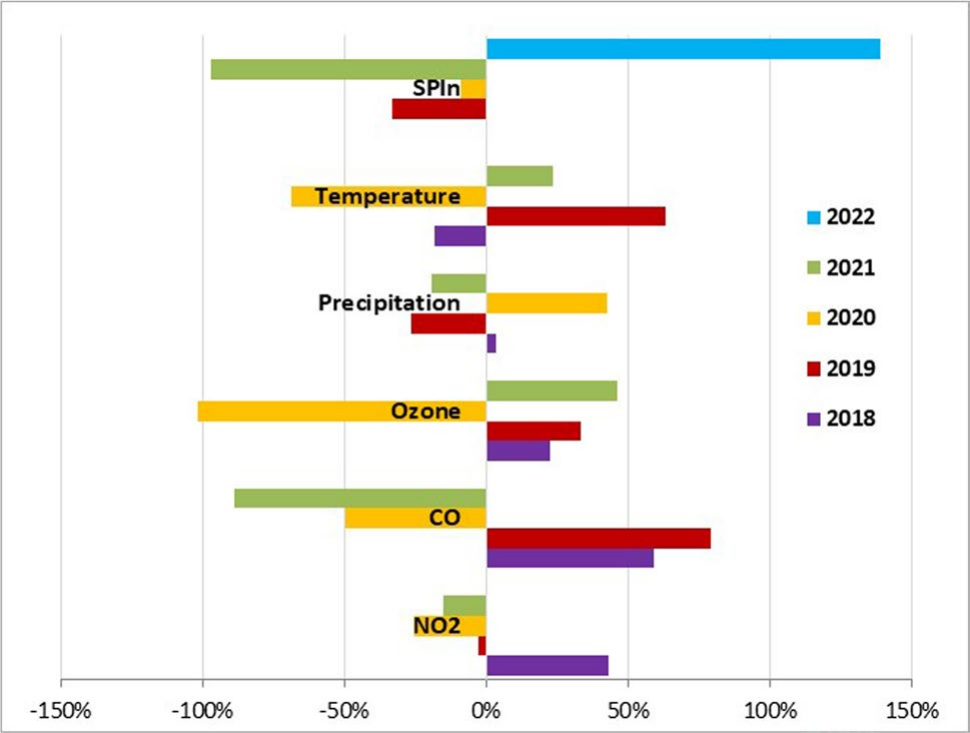
Increase/decrease in the levels of SPIn and selected environmental parameters in Bratislava in June over the individual years compared to the 4-year-average values (2018 − 2021)

To evaluate the influence of the conditions in the pre-season period on SPIn, we studied the selected weather factors and air pollutants. The weather and air pollution in June differed significantly over the studied years (Tables S1–2). The strongest effect on SPIn was attributed to precipitation and CO levels. We found that when precipitation in June 2020 was high (186% more than the long-term average, 1983‒2022, Meteorological observatory Bratislava – Mlynská dolina), the SPIn attained a very low level in 2021 (1484 pollen*day/m^3^). Additionally, when the mean daily values of sunshine and air temperature reached high values over the January–February (3.9 h and 3.9 °C in 2022 vs 2.6 h and 1.8 °C in 2021), the SPIn was also high (19,975 in 2022 vs 1484 pollen*day/m^3^ in 2021) (Fig. 3).

**Fig. 4:**
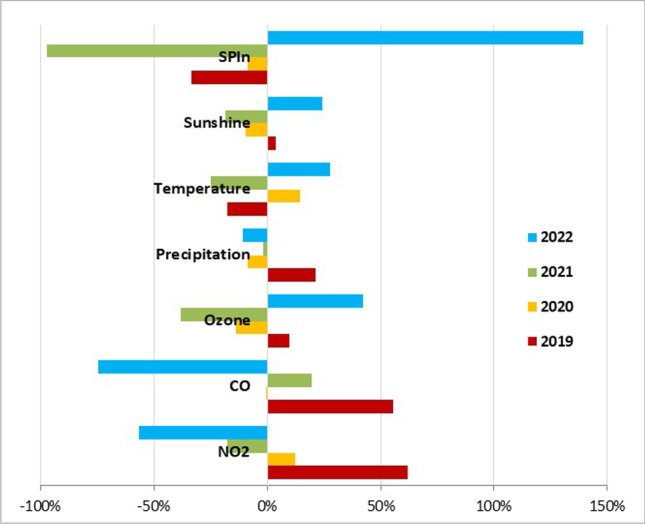
Increase/decrease in the in the average January‒February levels of SPIn and selected environmental parameters in Bratislava over the individual years compared to the 4-year-average values (2019 − 2022)

The birch SPIn in a particular pollen season also depends on the conditions in January − February of the same year when the plant dormancy ends. The strongest effect on SPIn was attributed to mean air temperature and sunshine. The parameters were compared to the average of the 4 analyzed years (2019–2022). We found that in 2022, when the levels of air temperature and sunshine were 28 and 24% higher, respectively, there was a 139% increase in SPIn (19,975 pollen/m^3^). On the other hand, a simultaneous decrease in air temperature (by 25%) and sunshine (by 19%) in 2021 led to an 97% lower value of SPIn (1484 pollen/m^3^) (Fig. 4).

**Fig. 5:**
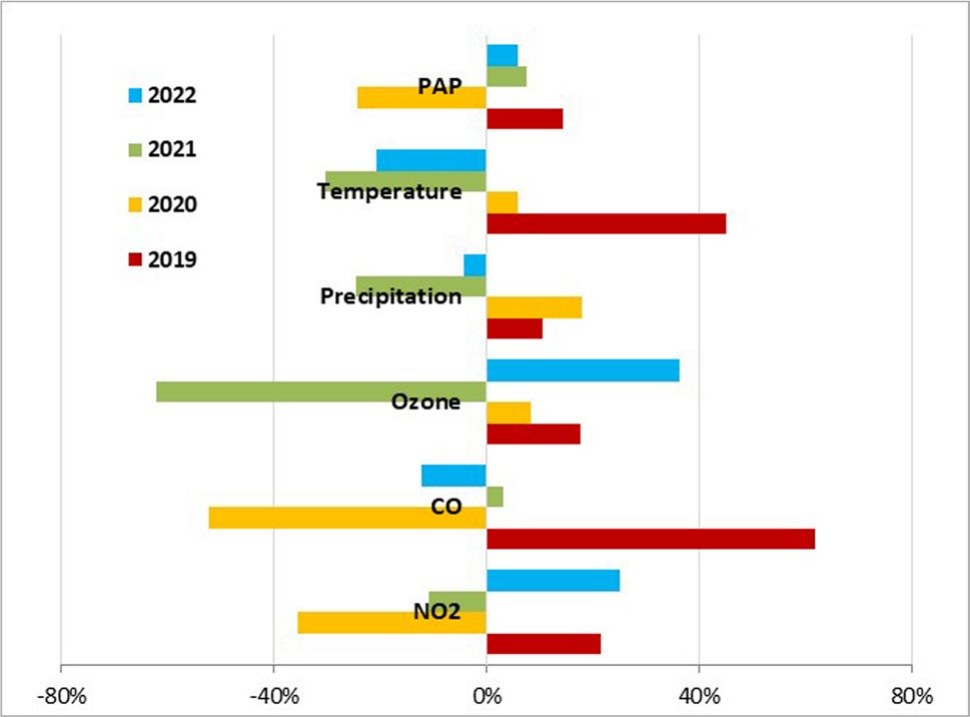
Increase/decrease in the levels of PAP and selected environmental parameters in Bratislava in March over the individual years compared to the 4-year-average values (2019 − 2022)

The conditions in March are one of the determining factors for the PAP of birch pollen in a particular pollen season. While the meteorological factors showed no association with PAP, gaseous pollutants (CO and NO_2_) had the strongest effect. The parameters were compared to the 4-year averages of 2019–2022. The above average CO (393.1 μg/m^3^) and NO_2_ (25.7 μg/m^3^) concentrations in March 2019 (62 and 21% higher, respectively) raised the mean PAP level by 14% (4.0 pg Bet v 1/pollen), while below average concentrations (52% less CO and 35% less NO_2_) in March 2020 lowered it by 24% (2.5 pg Bet v 1/pollen) (Fig. 5).

The conditions during the MPS influence the discharge of pollen from anthers and their airborne transport, Bet v 1 allergen production, and its release from pollen grains. The most influential meteorological parameters were air temperature, relative humidity, and precipitation, while the concentration of CO, NO_2_, and PM_10_ had the greatest impact among the air pollutants. The influence of individual environmental parameters on the airborne pollen and allergen concentrations and the PAP levels during the birch MPS were identified by multiple regression analysis (Table 2). Based on our results, relative air humidity and mean air temperature (1-day lag) were the most significant meteorological parameters influencing the airborne pollen concentration, whereas precipitation was crucial for allergen levels. Relative humidity and precipitation were negatively associated with pollen and allergen concentrations, respectively, while the relationship between air temperature and pollen levels was positive. Among the atmospheric pollutants, NO_2_ (1-day lag) and CO were significantly and positively interrelated with pollen and allergen levels, respectively. Furthermore, we observed a significant negative association between PM_10_ and allergen concentration. The air pollutants significantly related to the PAP were ozone and NO_2_ (1-day lag), though these associations were negative. Besides weather variables and air pollutants, Bet v 1 allergen was significantly and positively associated with pollen levels. Additionally, the results of the regression analysis show that both allergen and pollen levels were significantly higher in 2022 than in 2019, while in 2021, pollen levels were significantly lower compared to 2019.

**Table 2:** Significant environmental variables in multiple regression models for *Betula* pollen, Bet v 1 allergen, and pollen potency over the main birch pollen seasons, years 2019 − 2022

	Variables	*β* coeff	Std. error	*p*-value	*R*^2^
Pollen	Intercept	3.8402	0.6823	< 0.0001	0.4958
	RH	− 0.0168	0.0071	0.0199	
	T_mean_.lag1	0.0606	0.0284	0.0351	
	NO_2_.lag1	0.0504	0.0163	0.0024	
	year 2020	0.2723	0.4028	0.5004	
	year 2021	− 0.846	0.4029	0.0378	
	year 2022	1.4445	0.3987	0.0004	
Allergen	Intercept	− 353.8287	160.8853	0.0298	0.6512
	log(pollen)	98.701	12.6989	< 0.0001	
	P	− 7.1687	3.1422	0.0243	
	PM_10_	− 8.8814	2.0977	< 0.0001	
	CO	1.2386	0.4151	0.0034	
	year 2020	78.8506	60.6030	0.1957	
	year 2021	− 20.2466	68.6900	0.7687	
	year 2022	256.9595	71.8471	0.0005	
Pollen allergen potency	Intercept	2.9685	0.5988	< 0.0001	0.1432
	O_3_	− 0.0205	0.0084	0.0156	
	NO_2_.lag1	− 0.0404	0.0147	0.007	

*RH*, relative humidity; *T*_*mean*_, mean daily air temperature; *P*, precipitation; *PM*_*10*_, particulate matter ≤ 10 μm; *CO*, carbon monoxide; *O*_*3*_, ozone; *NO*_*2*_, nitrogen dioxide. *β* coefficient of the variable “year 2020” expresses the average difference between the values in 2020 and 2019 in this model, similarly for variables “year 2021” and “year 2022”

Environmental variables have explained 49.6 and 65.1% of the data dispersion for birch pollen and Bet v 1 allergen concentrations, respectively. However, for the PAP, air pollutants have explained only 14.3% of the total variance (Table 2).

## Discussion

Similarly to other researchers (e.g., Piotrowska and Kubik-Komar 2012; Grewling et al. 2012; Adams-Groom et al. 2022 and articles cited therein), we observed that birch pollen production is affected by meteorological conditions at different stages of plant development. In Bratislava, the SPIn was predominantly influenced by precipitation in June (1 year before pollination), when the formation of buds takes place, and air temperature and sunshine over January–February during the pollen development. Similarly to our results, Piotrowska and Kubik-Komar (2012) observed a negative association between total precipitation in June in the year preceding pollen release and birch SPIn in Lublin (Poland). They also found that lower SPIn was associated with lower daily air temperature in February. The negative influence of weather parameters in the year before pollination (May − June) on birch SPIn was also observed by Grewling et al. (2012) in Poznań (Poland); however, in this case, the average daily minimum temperature was responsible for the drop in SPIn. In contrast, the mentioned researchers noticed a negative relationship between precipitation and SPIn during February in the same year as pollination.

Besides meteorological parameters, air pollutants are also supposed to affect pollen production. We observed that raised SPIn levels were partially attributed to increased CO levels in June (1 year before pollination). It is in line with Jochner et al. (2013), who noted a negative association between pollen production and air pollution, albeit they considered NO_2_ the influential air pollutant.

Except for meteorological conditions, seasonal variability in pollen production may also correspond with the biological rhythms of the plants, as several researchers (e.g., Jato et al. 2002; Kubik-Komar et al. 2021) hint at the possibility of cyclic alternation (2- or 3-year fluctuation) in pollen output, with years of high and low annual totals of birch pollen concentrations. However, in our case, the 79% lower and 179% higher than the long-term average values of the birch SPIn in 2021 and 2022, respectively (2002 − 2022, pollen data of the Comenius University in Bratislava), were predominantly attributed to weather conditions during the birch pre-season period.

Besides SPIn, year-to-year fluctuations in SAIn and PAP levels were also found. Allergenic molecules are synthesized in pollen grains at the last stage of their maturation in the anthers, about a week before pollination (Buters et al. 2010). During this period, the traffic-related gaseous air pollutants, especially CO and O_3_ as known stressors for plants (Wang and Liao 2016; Hasan et al. 2021), can significantly affect the allergenic potential of pollen grains by altering their protein content (Beck et al. 2013; Plaza et al. 2016). Exposure of birch pollen even to low levels of CO and O_3_ was associated with enhanced allergenicity (Cuinica et al. 2015). This is in agreement with our findings, since we found that higher CO concentrations in March 2019 enhanced the expression of the Bet v 1 in birch pollen, manifested by raised mean and maximum daily PAP levels. On the contrary, the decrease in birch PAP in 2020 was associated with improved air quality, attributed especially to declined CO levels due to COVID-19 restrictions, which came into force on March 16 in the study area.

Additionally, the delay in birch pollen release from anthers, provoked by humid or cold weather conditions, leads to higher allergenic potential of pollen (Maya-Manzano et al. 2022). In this context we observed that the earlier onset of birch pollen season and less humid weather over the MPS in 2020 was accompanied by decreased PAP levels. In contrast, the rainy weather and increased airborne concentrations of CO and NO_2_ over the MPS in 2019 led to raised levels of PAP. Moreover, the high frequency of rainy days and drop in sunshine hours over the MPS in 2019 resulted in a shortened pollen season, as the pollination process can be limited due to such weather conditions (Dąbrowska-Zapart and Niedźwiedź 2022).

High daily pollen concentrations in the air during MPS are generally associated with warm and dry weather, resulting in favorable conditions for pollen liberation from anthers and their dispersion in the air (Kubik-Komar et al. 2019). This statement is consistent with the results of our study, as we observed a significant positive correlation between airborne birch pollen concentrations and air temperatures 1 day prior to pollen liberation and a negative relationship with relative humidity.

The number of allergenic molecules in the air depends primarily on the airborne pollen levels. The more pollen grains in the air, the higher the allergen concentration, as is confirmed by the results of our study. However, pollen concentrations of the causative plant taxa do not always correlate well with allergic symptoms, as is confirmed by several researchers (e.g., Buters et al. 2012; Ščevková et al. 2021). There may be several causes, but pollen fragmentation plays the most vital role. Rupture of pollen, whether due to osmotic shock caused by increased humidity (D’Amato et al. 2021) or electrical fields generated by lightning strikes in thunderstorms (Hughes et al. 2020), can lead to onset of severe pollen-related allergic conditions, such as asthma attacks and anaphylaxis. In the pollen fragmentation process, sub-micronic allergenic particles of 0.5 − 2.5 μm in diameter, which can, due to their small size, penetrate to lower airways and trigger allergic reactions in susceptible individuals, can be released into the air (D’Amato et al. 2016). Pollen disintegration, as well as the dispersion of pollen-related allergic fragments or free allergens in the air, is influenced by meteorological factors. Maya-Manzano et al. (2022) observed high Bet v 1 allergen levels during days with high humidity, when there was little to no airborne birch pollen. However, the regression analysis in our study showed that the amount of Bet v 1 allergen in the air decreases with rising precipitation, whereas air humidity was not influential. The reason is that both free allergens and allergen-containing pollen fragments are subject to the same physical laws as other airborne particles and are washed out of the atmosphere by precipitation (Pérez et al. 2009).

On the other hand, some discrepancies in the daily pollen and allergen concentrations during the MPS may be associated with non-local pollen incorporated in long-distance atmospheric transport. Airborne pollen grains transported over long distances usually lack allergens as these have been released in advance due to the stress conditions to which pollen is exposed during transport (Sofiev et al. 2013). Although tiny birch pollen grains (20 − 25 µm) are often involved in long-range atmospheric transports (Myszkowska et al. 2021), those recorded in the air of Bratislava are principally of local origin, as evidenced by the significant correlation between birch pollen and Bet v 1 allergen concentrations over the MPS, pre-peak, and post-peak periods.

Besides meteorological parameters, air pollutants were associated with day-to-day variation in airborne pollen and allergen concentrations. We noted the concurrent rising of (1) birch pollen and NO_2_ levels and (2) Bet v 1 allergen and CO levels over the MPS. Several researchers (e.g., Takahashi and Morikawa 2014; Zhao et al. 2017) recognize nitrogen dioxide as an air pollutant that positively regulates the growth of vegetative and generative structures of plants, resulting in enhanced biomass and pollen production. On the other hand, carbon monoxide is associated with stress-related defense processes in plants, leading to enhanced allergenic protein content in pollen grains (Žiarovská et al. 2013). However, in this study, the significant positive relationship between airborne birch pollen and NO_2_ or between Bet v 1 allergen and CO concentration can be more likely attributed to weather than pollutants, as there was also a significant positive association of air temperature with NO_2_ and CO levels in the studied area.

Based on our results, we can conclude that during the birch MPS, ozone and NO_2_ (a major pollutant involved in atmospheric ozone production) have a significant negative relationship with PAP. Similar results considering ozone were reported by other researchers, such as, e.g., Plaza et al. (2020). These negative relationships can be most probably attributed to meteorological factors such as temperature and solar radiation which affect formation of mentioned air pollutants (Coates et al. 2016). The tropospheric ozone, originating mainly from vehicles and industry, is formed by the reactions of NO_X_ with VOCs, which take place especially in hot sunny weather (Manisalidis et al. 2020).

The pollutants adhered to pollen can damage its surface, which is accompanied by an enhanced release of allergens (Vasilievskaya 2022). In contrast, we observed a significant negative correlation between allergen concentration and PM_10_ over the MPS. In this context, we hypothesize that a high concentration of solid particles (including PM_10_), which can attach to pollen exine in the air, causes pollen grains to become heavier, thus deteriorating their aerodynamic properties and increasing their sedimentation velocity (Hirose and Osada 2016). Alternatively, we assume that too many adhered particles to pollen grains may result in the plugging of pollen pores, thus preventing allergen liberation. However, further research is necessary to confirm or disprove these hypotheses.

## Conclusions

We observed significant seasonal fluctuations in the airborne levels of birch pollen and Bet v 1 allergen and birch PAP. We concluded that the resulting levels of SPIn, SAIn, and PAP are determined by a complex combination of factors interacting among themselves.

Considering SPIn, the most pronounced factors were precipitation and CO levels in June (1 year preceding pollination) when buds are formed and air temperature and sunshine hours over the January‒February period when the development of pollen grains continues after dormancy. Rainy weather in June was accompanied by a decrease in SPIn, whereas warm sunny weather in January‒February resulted in higher SPIn values.

The concentration of air pollutants in March, the time of pollen maturation, affected PAP levels. Higher CO and NO_2_ concentrations in March were associated with rising PAP levels and vice versa.

Day-to-day variations in pollen, allergen, and PAP levels during the MPS were principally attributed to weather. However, the allergen levels were also positively associated with pollen. The relationship between air temperature and pollen was positive, while the association between relative humidity and pollen was negative. A negative relationship between precipitation and allergen levels was also noted, the wash-out mechanism probably being responsible.

## Supplementary Information

Below is the link to the electronic supplementary material.

Supplementary file1 (DOCX 34 KB)

## Data Availability

All data generated or analyzed during this study are included in this published article.
