# Supplementary material for: Impact of meteorological parameters and air pollutants on airborne concentration of Betula pollen and Bet v 1 allergen
Source: Environ Sci Pollut Res Int. 2023 Aug 7;30(42):95438–48. doi: 10.1007/s11356-023-29061-z (PMC10482788; doi:10.1007/s11356-023-29061-z)
Supplement: Supplementary file 1 — Supplementary file1 (DOCX 34 KB) [file 11356_2023_29061_MOESM1_ESM.docx]

**Supplementary Materials**

*Impact of meteorological parameters and air pollutants on airborne concentration of Betula pollen and Bet v 1 allergen*

Jana Ščevková. Jozef Dušička. Eva Zahradníková. Regina Sepšiová. Jozef Kováč. Zuzana Vašková

**Table S1** The average monthly values of meteorological parameters monitored in Bratislava

| Year | Month | T_mean_  (C) | P  (mm) | RH  (%) | S  (h) |
| --- | --- | --- | --- | --- | --- |
| 2018 | VI | 20.5 | 103 | 72 | 8.0 |
|  | VII | 21.8 | 136.4 | 68 | 9.0 |
|  | VIII | 23.5 | 70.7 | 64 | 8.7 |
|  | IX | 17.4 | 183.1 | 74 | 7.0 |
|  | X | 13.5 | 25.8 | 73 | 4.9 |
|  | XI | 6.7 | 60.9 | 87 | 2.5 |
|  | XII | 1.9 | 161.3 | 87 | 1.1 |
| 2019 | I | 0.0 | 118.6 | 78 | 2.0 |
|  | II | 4.2 | 32.3 | 69 | 4.5 |
|  | III | 8.5 | 57.3 | 62 | 5.2 |
|  | IV | 12.0 | 39.0 | 60 | 6.9 |
|  | V | 12.6 | 260.6 | 75 | 4.3 |
|  | VI | 23.3 | 18.4 | 64 | 10.4 |
|  | VII | 22.1 | 81.1 | 59 | 8.8 |
|  | VIII | 22.5 | 109.3 | 66 | 7.7 |
|  | IX | 16.6 | 97.1 | 69 | 6.2 |
|  | X | 12.4 | 37.6 | 77 | 4.7 |
|  | XI | 8.2 | 115.2 | 86 | 1.6 |
|  | XII | 3.5 | 115.2 | 83 | 1.9 |
| 2020 | I | 0.4 | 27.0 | 90 | 2.2 |
|  | II | 6.2 | 58.8 | 65 | 3.6 |
|  | III | 7.0 | 67.9 | 58 | 6.0 |
|  | IV | 13.0 | 3.9 | 44 | 9.0 |
|  | V | 13.9 | 110.9 | 63 | 7.7 |
|  | VI | 18.9 | 213.5 | 75 | 6.9 |
|  | VII | 21.1 | 42.7 | 63 | 9.2 |
|  | VIII | 21.5 | 152.9 | 70 | 6.7 |
|  | IX | 17.2 | 51.8 | 71 | 6.6 |
|  | X | 10.9 | 304.6 | 86 | 2.9 |
|  | XI | 5.0 | 16.4 | 93 | 2.1 |
|  | XII | 3.1 | 90.6 | 94 | 1.0 |
| 2021 | I | 1.4 | 60.9 | 87 | 1.8 |
|  | II | 2.1 | 39.1 | 84 | 3.5 |
|  | III | 5.5 | 7.0 | 67 | 5.8 |
|  | IV | 8.5 | 95.3 | 67 | 6.3 |
|  | V | 13.3 | 139.8 | 74 | 6.7 |
|  | VI | 21.9 | 38.8 | 62 | 11.0 |
|  | VII | 22.7 | 80.6 | 64 | 9.9 |
|  | VIII | 19.3 | 151.8 | 76 | 6.5 |
|  | IX | 17.1 | 194.5 | 72 | 6.4 |
|  | X | 10.6 | 32.6 | 79 | 5.4 |
|  | XI | 5.6 | 87.9 | 91 | 1.9 |
|  | XII | 2.0 | 82.7 | 95 | 1.4 |
| 2022 | I | 2.3 | 30.6 | 83 | 2.9 |
|  | II | 5.3 | 48.5 | 72 | 4.8 |
|  | III | 5.9 | 36.1 | 49 | 7.2 |
|  | IV | 9.5 | 38.4 | 66 | 5.9 |
|  | V | 17.3 | 102.4 | 70 | 8.5 |

*T_min_* - minimum air temperature. *T_mean_* - mean air temperature. *T_max_* - maximum air temperature. *P* – precipitation (sum of daily totals). *RH* – relative air humidity

**Table S2** The average monthly concentrations of air pollutants monitored in Bratislava

| Year | Month | PM_10_ (µg/m^3^) | PM_2.5_ (µg/m^3^) | O_3_  (µg/m^3^) | CO (µg/m^3^) | NO_2_ (µg/m^3^) |
| --- | --- | --- | --- | --- | --- | --- |
| 2018 | VI | 20.3 | 10.2 | 83.7 | 310.9 | 18.3 |
|  | VII | 19.9 | 10.1 | 89.8 | 234.3 | 18.2 |
|  | VIII | 22.3 | 10.8 | 86.1 | 271.9 | 23.5 |
|  | IX | 22.0 | 11.2 | 70.2 | 401.4 | 26.8 |
|  | X | 35.3 | 20.6 | 52.0 | 414.6 | 28.1 |
|  | XI | 32.1 | 24.6 | 25.8 | 597.0 | 28.3 |
|  | XII | 25.0 | 21.8 | 29.3 | 496.3 | 27.1 |
| 2019 | I | 22.0 | 18.3 | 41.6 | 433.5 | 28.0 |
|  | II | 28.8 | 22.0 | 46.1 | 503.2 | 31.9 |
|  | III | 18.8 | 11.6 | 66.0 | 393.1 | 25.7 |
|  | IV | 28.0 | 17.2 | 76.9 | 387.5 | 23.4 |
|  | V | 14.4 | 8.8 | 69.9 | 354.0 | 19.3 |
|  | VI | 23.8 | 11.5 | 85.3 | 324.8 | 16.0 |
|  | VII | 18.8 | 8.7 | 84.7 | 293.2 | 17.1 |
|  | VIII | 18.4 | 9.1 | 77.7 | 329.6 | 18.8 |
|  | IX | 17.6 | 8.3 | 57.4 | 355.7 | 20.6 |
|  | X | 26.2 | 16.5 | 41.5 | 419.2 | 25.3 |
|  | XI | 19.6 | 14.0 | 28.5 | 390.9 | 23.0 |
|  | XII | 22.2 | 15.8 | 33.5 | 383.2 | 24.3 |
| 2020 | I | 30.1 | 24.1 | 26.0 | 493.1 | 29.3 |
|  | II | 13.5 | 10.8 | 55.2 | 283.5 | 20.4 |
|  | III | 22.7 | 15.5 | 64.9 | 303.7 | 20.7 |
|  | IV | 23.7 | 16.0 | 81.0 | 288.4 | 19.7 |
|  | V | 15.5 | 10.6 | 75.1 | 247.2 | 15.1 |
|  | VI | 15.0 | 9.8 | 65.7 | 236.4 | 14.8 |
|  | VII | 18.1 | 10.7 | 71.1 | 233.6 | 16.9 |
|  | VIII | 19.3 | 11.1 | 70.8 | 264.4 | 18.7 |
|  | IX | 21.0 | 9.4 | 56.5 | 295.7 | 21.0 |
|  | X | 17.4 | 9.2 | 34.8 | 324.0 | 17.6 |
|  | XI | 225.1 | 25.1 | 21.3 | 218.6 | 17.2 |
|  | XII | 20.1 | 15.1 | 24.0 | 412.7 | 16.4 |
| 2021 | I | 18.9 | 14.7 | 32.9 | 389.6 | 20.2 |
|  | II | 33.7 | 24.0 | 39.8 | 450.5 | 24.1 |
|  | III | 23.9 | 16.4 | 56.3 | 347.4 | 22.9 |
|  | IV | 17.7 | 11.6 | 69.4 | 274.7 | 15.5 |
|  | V | 9.6 | 5.6 | 67.6 | 233.4 | 14 |
|  | VI | 21.6 | 15.7 | 87.2 | 209.6 | 15.4 |
|  | VII | 17.6 | 12.2 | 81.2 | 201.9 | 13.6 |
|  | VIII | 11.8 | 7.0 | 67.0 | 235.5 | 13.8 |
|  | IX | 17.0 | 9.1 | 63.0 | 289.9 | 20.4 |
|  | X | 23.6 | 14.9 | 45.8 | 337.1 | 21.0 |
|  | XI | 21.2 | 16.4 | 28.3 | 345.8 | 21.3 |
|  | XII | 17.3 | 14.8 | 29.5 | 359.8 | 21.7 |
| 2022 | I | 16.2 | 12.9 | 43.2 | 301.2 | 18.7 |
|  | II | 13.1 | 9.0 | 55.3 | 279.2 | 17.6 |
|  | III | 29.8 | 21.8 | 68.3 | 335.4 | 26.0 |
|  | IV | 14.9 | 10.4 | 63.9 | 240.7 | 15.4 |
|  | V | 16.2 | 9.6 | 78.6 | 205.2 | 16.1 |
